# Supplementary material for: Patterns of Intron Gain and Loss in Fungi
Source: PLoS Biol. 2004 Nov 30;2(12):e422. doi: 10.1371/journal.pbio.0020422 (PMC532390; doi:10.1371/journal.pbio.0020422)
Supplement: Table S1 — Also available at http://genes.mit.edu/NielsenEtAl/. (4.3 MB ZIP). [file pbio.0020422.st001.zip › NielsenEtAl/html/403.html]

AN9469.1.NCU07852.1.MG05282.1.FG04125.1


```
 CLUSTAL W (1.82) Multiple Sequence Alignments - Introns Inserted


Sequence 1: NCU07852.1	183 aa
Sequence 2: MG05282.1	183 aa
Sequence 3: FG04125.1	179 aa
Sequence 4: AN9469.1	166 aa
Alignment Length: 183 aa
Number Identitical Residues: 92 aa
Alignment Score (without introns) 4621


MG05282.1 	MSQTVGL0TRLAYSRAWHHISASAPHQVLSTVKSPPEAVTPPSLGRLASRIATILMGKHK
NCU07852.1	MSQTVGA0TRLAYSRVWHHISAVTPHPTLSTIKAPSEAITPPSLGRLASRIATILMGKHK
FG04125.1 	MSQVLGL0TKLAYSRVWHGVSASAPHATLSTQPG----VTPPSLGRLASRIAVLLMGKHK
AN9469.1  	MSQTVGK0TRLAYSRAWHLVDVGS---------------DGRSLGRLASSIALILMGKNK
          	***.:*  *:*****.** :.. :                  ******* ** :****:*

MG05282.1 	PIWDPSTDCGDYVVVTNCAALYTTGMKKYRKTYYRHNTRPGSLKSVTMDVLMDKFGGSEV
NCU07852.1	PIWDPSTDCGDYVVVTNCAGLYTTGHKKWRKTYYRHNTRPGSLQAITMDALMEKHGGAEV
FG04125.1 	PIFDPSTDCGDYVVVTNCAALHTTGRKKWQKPYYRHTTRPGSLKTVTMDVLMEQHGGSEV
AN9469.1  	PIYDPSTDCGDYVVAVGCHDLHTTGKKRFQKKYYTHTTRPGSLRSMTMDKMFEKWGGGEV
          	**:***********...*  *:*** *:::* ** *.******:::*** :::: **.**

MG05282.1 	LRKAVSGMLPKNRLRDKRLARLKA~FEGEAHPYKRNIVRFGDKTVGRPGWEEAVKAIRAA
NCU07852.1	LRKAVSGMLPKNRLRDKRLARLKA~FEGDAHPYKENLVRFGGKVVGAPGWEEAVKAIREA
FG04125.1 	LRKAVSGMLPKNRLRDKRLARLKA~FEGDAHPYKQNLIRFSGVPVGKPGWEKAVEKVREG
AN9469.1  	LRRAVRGMLPKNRLRDKRLARLKT1FEGVDHPYKDNIVRIG-APSNIGALPEVKAAFQEA
          	**:** *****************: ***  **** *::*:.    .  .  :.   .: .

MG05282.1 	DMNRL
NCU07852.1	DMERL
FG04125.1 	DKKRL
AN9469.1  	KTSQ-
          	. .:
```
